# Validity of Alfred Step Test Exercise Protocol (A‐STEP) as a Surrogate VO_2Max_ Cardiopulmonary Exercise Test (CPET) to Cycle Ergometry in Adults With Cystic Fibrosis

**DOI:** 10.1155/carj/9062245

**Published:** 2026-03-19

**Authors:** Brenda Button, Mahesh Dharmakumara, Lisa Wilson, Nic Parry, Faizel Hartley, Brigitte Borg, Dominic T. Keating

**Affiliations:** ^1^ The Alfred, Melbourne, Australia, alfred.org.au; ^2^ Faculty of Medicine, Nursing and Health Sciences, Monash University, Melbourne, Australia, monash.ac.za

## Abstract

**Background:**

The Alfred Step Test Exercise Protocol (A‐STEP) and feasibility study were previously published. The aim here was to determine the validity of the A‐STEP compared to cycle ergometry (CPET) in adults with CF.

**Methods:**

The A‐STEP and CPET were carried out in random order 2 weeks apart. A wearable, portable metabolic system was used to measure breath‐by‐breath and minute‐by‐minute sampling of O_2_, CO_2_, heart rate, and VO_2_. The main outcome measures were VO_2max_ and HRmax.

**Results:**

Seven stable‐state adults (3 male) on CFTR modulator therapy with a mean (SD) and range of age 38.2 (13.4) 26–64 years; height 169.9 (10.9) 149.7–185.3 cm; BMI 22.8 (2.10) 19.5–28 kg/m^2^; FEV1 79.4 (18.9) 38.0–106.0; and FVC 95.1 (16.7) 63.0–114.0 percent predicted (pp) completed both A‐STEP and CPET. The VO_2Max_ had high correlation and good agreement between the A‐STEP 31.3 (5.9) and CPET 29.8 (6.2) mL/min/kg, *r* = 0.88. The HR_MAX_ was strongly correlated with the A‐STEP 174 (17) bpm and 95.7 (7.4) pp versus 168 (15) bpm and 92.4 (5.3) pp with *r* = 0.92 and 0.86, respectively. The SpO_2Nadir_ for A‐STEP was 91.0 (4.0) and CPET 92.0 (3.3), *r* = 0.82. The VO_2_ at the anaerobic threshold (VO_2@AT_) occurred significantly earlier for the CPET at 1021 (260) versus A‐STEP 1361 (234) mL/minute, *p* < 0.05. The VE_Max_ for CPET was 84.1 (18.8) and A‐STEP 73.5 (15.8) L/minute, *p* < 0.05. The AWESCORE also ensured baseline stability. The number of levels completed during the A‐STEP was 10.7 (12.9) ranging from 9 to 15.

**Conclusion:**

The A‐STEP may be a portable, valid surrogate to cardiopulmonary exercise testing using cycle ergometry.

## 1. Introduction

### 1.1. Background/Rationale

Cardiorespiratory fitness in cystic fibrosis (CF) is associated with better health outcomes [1]. Cardiopulmonary exercise testing (CPET) on a cycle ergometer is the gold standard exercise capacity test [2] but is not available at all CF centers. It requires expensive equipment and experienced technicians to carry out and analyze the results of the test. Results are not immediately available in real time in the clinical setting for exercise prescription or the identification of the need for supplemental oxygen in those who desaturate during exercise.

We previously studied the three‐minute step test (3MST). This was carried out on a 15 cm step and externally paced with a metronome at a cadence of 30 steps per minute for three minutes [3]. It was found to be useful to determine whether people with CF (pwCF) with advanced lung disease experienced oxygen desaturation during exercise and predicted the requirement of more medical treatment in the following 12 months. A floor effect was found in those with low lung function, and a ceiling effect was found in individuals with mild to moderate respiratory disease [3, 4]. Therefore, the 3MST was unsuitable for use as a maximal exercise capacity test across the lifespan resulting in the recommendation from the ECFS Exercise Working Group that the 3MST no longer be used as an exercise capacity test in CF [5].

In a recent systematic review of the clinimetric properties of field tests in CF, the incremental step test (IST), a modified version of the 3MST, was compared with other step tests used in CF [6]. It utilized the same step height of 15 cm but the stepping cadence and duration of the test were different. Subjects began stepping at a rate of 20 steps per minute for 2 min which was increased by 10 steps per minute for a test duration of 6 min. The IST demonstrated a ceiling effect, making it unsuitable for use as an exercise capacity test in CF [7, 8].

While the modified shuttle test has been recommended in CF, it requires a larger space where pwCF can walk or run up and down the 10 m length of the test safely. Not all clinics have a suitable space [4]. Over the last decade, we identified the need for an incremental maximal exercise test for pwCF that is able to be used in a standard outpatient clinic room. Test conditions should also comply with modern infection control guidelines. Further, we wished to develop a test that could be used across the lifespan in CF from childhood to older age and across the range of height, BMI, and lung function. Therefore, we set about developing a CF‐specific incremental maximal exercise step test using the CPET protocol and attributes as our guide. The Alfred Step Test Exercise Protocol (A‐STEP) was developed over a number of years with the assistance of pwCF [9]. This was followed by a feasibility study across the lifespan including a pediatric sample from Adelaide Women’s and Children’s Hospital, South Australia [10]. The validity of the A‐STEP has not been tested to date.

### 1.2. Objectives

The aim of this study was to determine the validity of step ergometry using the A‐STEP as a comparable incremental maximal exercise capacity test to CPET using standard cycle ergometry, in a broad range of adults with CF.

## 2. Materials and Methods

### 2.1. Study Design

The design was a prospective single‐center observational study, approved by the Human Research Ethics Committee of Alfred Health (205/16) and Monash University. Written informed consent was obtained from all study volunteers. Recruitment and data collection were carried out between February 2023 and November 2024. Data analysis occurred between January 2025 and April 2025.

### 2.2. Participants

Participants for the study were adults with a confirmed diagnosis of CF based on elevated sweat chloride and compatible genotype aged between 26 and 64 years. They were prospectively recruited and could have any level of fitness and exercise engagement. Eligible participants were required to be clinically stable at the time of recruitment, assessment, randomization, and testing.

Participants were excluded if they were pregnant; waitlisted for lung transplant or had received a lung transplant; had musculoskeletal conditions that would limit test performance; took medication known to impair exercise; had significant cardiac comorbidities; and had significant liver disease or treatment with intravenous antibiotics in the 4 weeks before starting the study. Seventy‐five subjects were potentially eligible; 50 were examined for eligibility, 25 were confirmed as eligible, and seven agreed to participate in the study and attend the laboratory on two separate days.

All participants attended Alfred Health for two study visits within a 1‐month period 2 weeks apart. They each carried out two alternate maximal exercise capacity tests using the A‐STEP and CPET protocols. The two tests were carried out in the Lung Function and Scientific Testing Laboratory of Alfred Health. The order of tests was determined using a computer‐generated sequence with random allocation stored securely. Participants were screened before each of the exercise tests to ensure clinical stability using spirometry and the short‐form, reliable, and validated wellness test, the AWESCORE [11]. They were required to have < 10% variation in FEV1 percent predicted (pp) from baseline in the preceding 6 months and similar AWESCORE totals at each of the two visits and no new respiratory symptoms.

### 2.3. Information Provided Before the Test

Before the first test, participants were provided with detailed verbal and written information about the requirements of the study days. These included suitable exercise clothing and footwear and the importance of being well hydrated on each testing day. They were instructed not to eat a full meal (including coffee) for 2 h before testing. Blood sugar levels were measured before the test if they had CFRD. Each subject was reviewed by a CF physician before the first test for suitability to carry out the two maximal exercise capacity tests. Both tests were carried out at the same time of day.

### 2.4. Equipment

#### 2.4.1. COSMED K5 Wearable Metabolic System

A wearable portable metabolic system (using the COSMED K5 system) [12] includes heart rate chest belt for breath‐by‐breath gas sampling including CO_2_ and O_2_ with wireless connectivity via a user‐friendly interface. Minute‐by‐minute heart rate, oxygen saturation, and measurements of VO_2_ were also undertaken.

### 2.5. Protocols

#### 2.5.1. A‐STEP

The A‐STEP [9] was carried out on a single portable 20 cm nonslip exercise step (Bodyworx Aerobic) for stepping up and down. There was an incremental stepwise intensity of step frequency starting at a cadence of 18 steps per minute (level 1) up to a maximum of 48 steps per minute (level 16). At the end of each minute, the cadence increased by 2 steps per minute. Participants practiced stepping up and down to the beat of the metronome and how to change leading legs to avoid unilateral leg fatigue before the test. The A‐STEP was externally paced using a digital metronome app freely available online (DenciSoft Circuit Timer Metronome App) for use on an iPhone, iPad, or Apple computer. The incremental cadence was set up for the A‐STEP and is available in the online protocol. Participants were invited to perform the A‐STEP until volitional exhaustion aiming for 90% of HRmax for age (HRmax; 220—age), and 9 or 10/10 on the modified Borg score for dyspnea and/or leg fatigue. The aim was for most subjects to complete maximal tests in eight to 12 min. The maximum time for very fit and elite athletes was 16 min. Level 16 requires participants to run fast up and down the steps.

#### 2.5.2. CPET

CPET was carried out according to the Godfrey protocol on a stationary cycle ergometer (Ergoline Ergoselect 100) with electronically controlled loading. Participants were invited to perform cycle ergometry until volitional exhaustion. There was an incremental stepwise intensity of pedaling load [2]. The test was completed as per the ATS/ACCP guidelines with 12‐lead ECG. A 10‐ or 15 W incremental selection protocol was used depending on lung function and reported baseline fitness of each subject tested [13, 14]. Resting measurements were made for 2 min before the test and followed by unloaded pedaling to the start of the test. The load was then increased every minute with measurements of VO_2_, SpO_2_, and HR recorded during the last 15 s of each stage [2]. Pedaling cadence was maximized at 60 revolutions per minute (rpm). During the test, a maximal symptom‐limited incremental CPET workload was progressively increased with the work rate increment aimed to bring the subject to volitional exhaustion in 10–12 min [13–17].

#### 2.5.3. Interpretation of Results

To interpret the results of exercise testing in pwCF, all factors that contribute to exercise capacity need to be considered. These included exercise fitness, severity of lung damage (FEV_1_), degree of bronchiectasis as evidenced on high‐resolution computer tomography (HRCT), as well as motivation and habitual exercise needed to be considered [18]. The same computerized K5 equipment was used for breath‐by‐breath analysis and minute‐by‐minute heart rate, oxygen saturation, and measurements of VO_2_ as described for the A‐STEP.

### 2.6. Main Outcome Measures

The primary outcome measures for the two maximal tests included peak oxygen uptake (VO_2_max) and peak heart rate (HRmax). Subjects were encouraged to achieve at least 90% of their HRmax through standardized encouragement during the test.

### 2.7. Oximetry

Oximetry (Nonin 7500), including oxygen saturation and heart rate, was measured and recorded at rest before the test; at the end of each level during the test; and at the end of each minute of the post‐test recovery period lasting 5 min.

### 2.8. Blood Pressure

Blood pressure (Phillips SureSigns) was measured before and at the end of the test as well as at the end of the five‐minute recovery period during the A‐STEP. It was not possible to measure after each level while stepping up and down during the test. Minute‐by‐minute manual BP was carried out by a medical doctor as per usual protocol during CPET.

### 2.9. Modified Borg Score

The modified Borg score 0–10/10 (denoting a maximal effort) was used to ascertain the level of dyspnea and leg fatigue at rest before the test; at the end of each minute during the A‐STEP; at the end of each of the 5‐min resting phase; and at the end of the test. During the last 10 s of both tests, dyspnea was referred to as “lungs,” leg fatigue was referred to as “legs,” and subjects pointed at the appropriate Borg score number selected from the enlarged laminated poster provided. Before starting each test in order to achieve a maximal test, subjects were encouraged to aim for 9 or 10/10 for dyspnea or leg fatigue (or both).

### 2.10. Spirometry

At each visit before exercise testing, baseline spirometry was performed (PlatinumDL, MGC Diagnostics, USA) according to ATS/ERS Spirometry Standards Guidelines 2003, using reference values derived from the European Respiratory Society Global Lung Function Initiative (GLI 2012).

### 2.11. AWESCORE

Subjects completed an Alfred Wellness Score (AWESCORE) a 10‐point visual analog scale (VAS) from 0 (least well) to 10 (most well). The AWESCORE consisted of two questions for each of the five domains comprising wellness including respiratory, physical, nutritional, psychological, and general health including sleep (amount and quality). Each of the 10 questions included descriptive anchors at 0 (least well) and 10 (most well) for each domain item. PwCF was asked to circle the number on each of the items that applied to them at the time of testing. Once completed, the 10 items were summated providing the total AWESCORE. The higher the score, the greater the perception of wellness. Spirometry and AWESCORE results were used to determine lung health and quality of life for each individual tested and to determine that each participant was clinically stable and at baseline state before each of the two tests [11].

### 2.12. Statistical Analysis

Metabolic and ventilatory exercise data were averaged using 20‐s breath‐by‐breath data. Descriptive data and results were expressed as mean ± SD and range. The comparison of A‐STEP versus CPET maximal exercise parameters was performed using a paired *t*‐test following the Shapiro–Wilk normality test.

Reliability and agreement were assessed by Bland–Altman plots and calculating the intraclass correlation coefficient (ICC) with 95% confidence intervals (CI) using absolute agreement based on a two‐way mixed‐effects model. For comparison with other published studies [19], Pearson’s correlation coefficient (*r*) was also performed. The statistical significance level for all analyses was set at a *p* value < 0.05 and performed using commercial statistical software (SPSS Version 23, Chicago, IL).

## 3. Results

Seven adults (3 male) with CF across the range of age, height, body weight, and lung function participated in this study. Patient characteristics are included in Table 1. All seven adults completed both A‐STEP and CPET in random order with complete analysis presented in Table 2. The primary outcome, VO_2Max_, had high correlation and good agreement between the A‐STEP 31.3 vs 29.8 mL/min/kg: *r* = 0.88. The HR_MAX_ was also strongly correlated with the A‐STEP with *r* = 0.92. The SpO2_Nadir_ for A‐STEP and CPET also correlated closely with *r* = 0.82. The oxygen consumption rate (VO_2_) at the anaerobic threshold (VO_2@AT_) occurred significantly earlier for CPET at 1021 (260) mL/minute versus A‐STEP at 1361 (234) mL/minute with *p* < 0.05. VE_Max_ for CPET was 84.1 (18.8) and for A‐STEP was 73.5 (15.8) L/min with *p* < 0.05 (Table 2; Figure 1). All seven subjects were well established and stable on elexacaftor–tezacaftor–ivacaftor, and most had been on earlier versions of CFTR modulators for at least 2 years. They were all clinically stable and at baseline, based on lung function and the AWESCORE. The higher the AWESCORE, the greater the sense of wellness. The following scores were recorded by the seven participants before testing demonstrating stability: test day (1) and test day (2): 80 78; 91 92; 75 78; 81 81; 88 88; 90 90; 71 74. The mean (SD) number of levels completed during the A‐STEP was 10.7 (12.9) ranging from 9 to 15.

**TABLE 1:** Subject demographics.

		**Mean ± SD**	**Range**

*n*	#	7	4F|3M

Age	yrs	38.2 ± 13.4	26.1–64.2

Height	cm	169.9 ± 10.9	149.7–185.3

Weight	kg	66.2 ± 10.4	53.5–83.9

BMI	kg/m^2^	22.8 ± 2.1	19.5–26.0

FEV_1_	%Pred	79.4 ± 18.9	38.0–106.0
	*z*‐score	−1.66 ± 1.52	−4.98–0.44

FVC	%Pred	95.1 ± 16.7	63.0–114.0
	*z*‐score	−0.45 ± 1.34	−3.06–1.04

FEV_1_/FVC	Ratio	0.68 ± 0.08	0.50–0.77
	*z*‐score	−1.94 ± 0.93	−3.69–−0.92

SpO_2 Rest_	%	97.4 ± 1.8	94.0–99.0

**TABLE 2:** Comparison of A‐STEP vs. CYCLE maximal cardiopulmonary exercise parameters (mean ± SD, range).

			A‐STEP		CPET		ICC (95% CI)	Pearson’s *r*
			Mean ± SD	Range	Mean ± SD	Range		
Protocol	Workload_Max_	Watts	—	—	133 ± 31	80–165	—	—
		%Pred	—	—	81.7 ± 20.0	58.0–107.0	—	—
	Cadence_Max_	Steps/min	37 ± 4	34–46	—	—	—	—
	Ex duration	Mins	10.6 ± 2.1	9.00–15.00	9.7 ± 1.0	8.00–11.00	0.66 (−0.49, 0.94)	0.69
	Borg_Modified_	Dyspnea	7.9 ± 2.1	4.0–10.0	7.7 ± 2.8	2.0–10.0	0.92 (0.49, 0.99)	0.86^#^
		Leg fatigue	8.6 ± 1.3	7.0–10.0	9.7 ± 0.5^∗^	9.0–10.0	0.23 (−0.68, 0.81)	0.31

Metabolic	VO_2Max_	mL/min/kg	31.3 ± 5.9	23.0–39.1	29.8 ± 6.2	20.4–36.9	0.93 (0.65, 0.99)	0.88^#^
		mL/min	2066 ± 452	1293–2555	1971 ± 479	1151–2420	0.97 (0.81, 1.00)	0.95^#^
		%Pred	76.3 ± 13.9	61.5–95.8	72.2 ± 12.3	56.4–86.1	0.93 (0.58, 0.99)	0.90^#^
	VCO_2 Max_	mL/min/kg	35.0 ± 7.4	23.6–44.3	37.4 ± 9.3	23.5–46.7	0.93 (0.61, 0.99)	0.91^#^
		mL/min	2352 ± 686	1327–3059	2476 ± 699	1325–3223	0.96 (0.79, 0.99)	0.93^#^
	RER_Max_	—	1.16 ± 0.13	1.02–1.31	1.25 ± 0.09	1.15‐1.35	0.66 (−0.32, 0.94)	0.67

Cardiac	HR_Max_	bpm	174 ± 17	146–193	168 ± 15	149–188	0.93 (0.51, 0.99)	0.92^#^
		%Pred	95.7 ± 7.4	86.1–104.6	92.4 ± 5.3	84.1–99.1	0.85 (0.15, 0.98)	0.86^#^
	VO_2_/HR_Max_	mL/beat	12.0 ± 2.8	8.2–15.5	11.8 ± 2.5	7.7–14.6	0.97 (0.84, 1.00)	0.94^#^
		%Pred	78.3 ± 13.3	58.0–94.0	76.4 ± 10.7	60.0–88.0	0.93 (0.63, 0.99)	0.88^#^
	BP systolic_Max_	mmHg	159.6 ± 13.7	152.0–190.0	149.6 ± 16.3	130.0–170.0	0.64 (−0.42, 0.93)	0.55
	BP diastolic_Max_	mmHg	75.4 ± 9.6	66.0–91.0	81.0 ± 10.8	63.0–92.0	0.62 (−0.63, 0.93)	0.49

Ventilatory	VE_Max_	L/min	73.5 ± 15.8	49.6–95.3	84.1 ± 18.8^∗^	58.3–103.3	0.87 (−0.12, 0.98)	0.91^#^
		%Pred	79.1 ± 19.4	53.8–110.7	88.6 ± 18.2^∗^	67.3–115.7	0.91 (−0.02, 0.99)	0.93^#^
	VT_Max_	L	1.97 ± 0.74	1.07–2.78	2.24 ± 1.05	0.95–3.60	0.94 (0.63, 0.99)	0.96^#^
	RF_Max_	Breaths/min	41.6 ± 9.6	29.0–53.0	44.5 ± 12.7	25.6–62.7	0.95 (0.70, 0.99)	0.97^#^
	VE/VCO_2Slope_	—	27.8 ± 5.8	22.1‐39.7	25.9 ± 6.3	19.4–37.8	0.90 (0.48, 0.98)	0.84^#^
	VO_2@AT_	mL/min/kg	20.7 ± 3.0	18.1–25.9	15.4 ± 2.8^∗^	9.6–17.6	0.23 (−0.27, 0.76)	0.33
		mL/min	1361 ± 234	1037–1719	1021 ± 260^∗^	544–1267	0.57 (−0.21, 0.92)	0.76^#^
		%Pred_VO2_	51.2 ± 12.3	37.7–76.8	37.1 ± 5.2^∗^	30.4–42.8	0.27 (−0.38, 0.81)	0.44
	VE/VCO_2Max_	—	31 ± 5	25–41	34 ± 6	27–42	0.81 (0.11, 0.97)	0.74
	SpO_2Nadir_	%	91.0 ± 4.0	84.0–95.0	92.0 ± 3.3	86.0–95.0	0.89 (0.42, 0.98)	0.82^#^

*Note:* Intraclass correlation coefficient (ICC).

^∗^ *p* < 0.05 paired *t*‐test.

^#^ *p* < 0.05 Pearson’s correlation.

FIGURE 1Bland–Altman plots of main exercise parameters (⸻ mean difference, ‐‐‐ limits of agreement). (a) Ex duration (mins). (b) VO_2Max_ (mL/min/kg). (c) HR_Max_ (bpm). (d) VE_Max_ (L/min). (e) VO_2@AT_ (mL/min/kg). (f) RER_Max_ (−).

**Figure:**
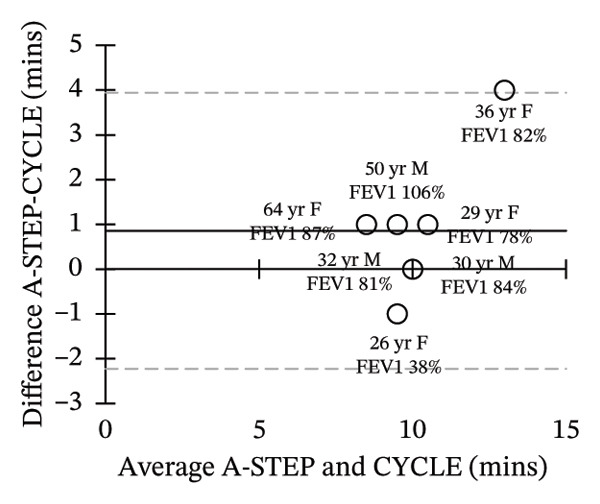
(a)

**Figure:**
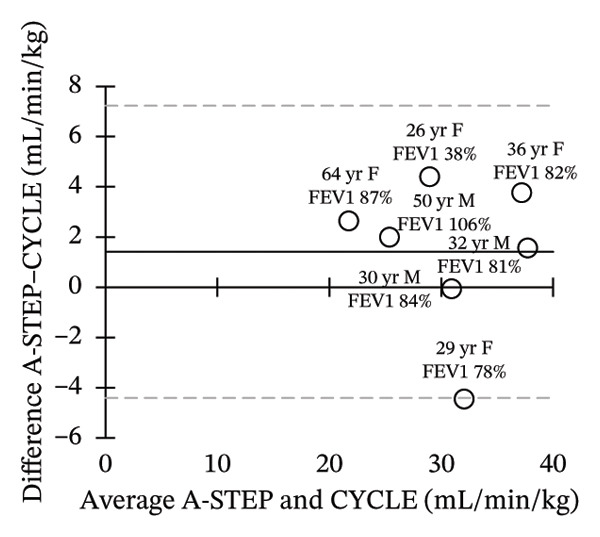
(b)

**Figure:**
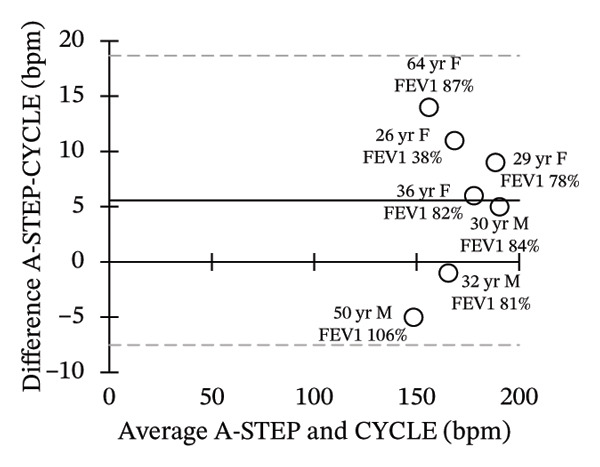
(c)

**Figure:**
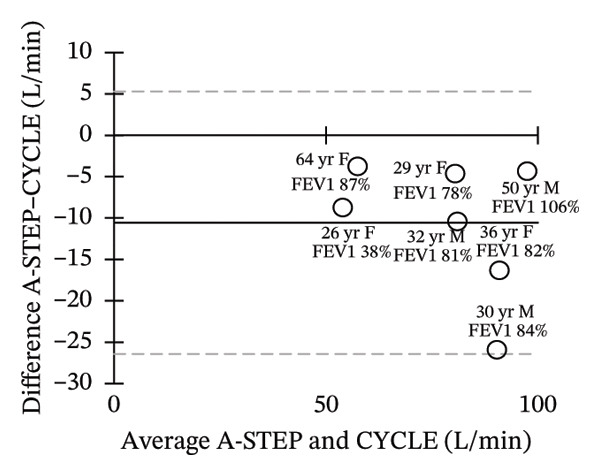
(d)

**Figure:**
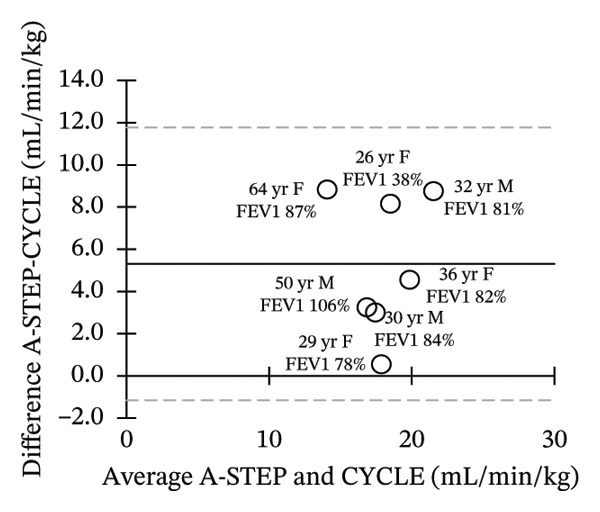
(e)

**Figure:**
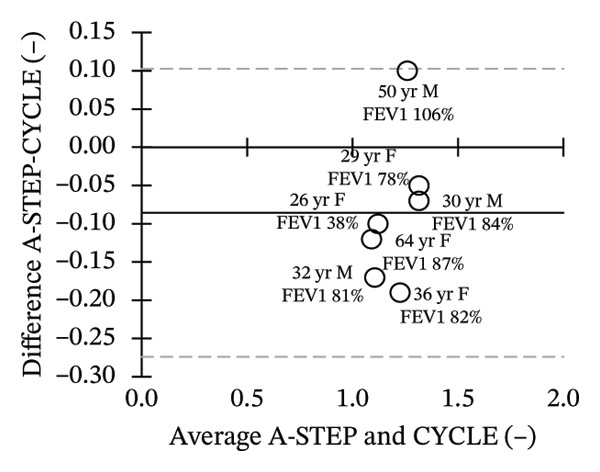
(f)

## 4. Discussion

In this pilot study, the A‐STEP achieved comparable exercise times to standard CPET. VO_2Max_, HR_Max_, RER_Max_, and SpO_2Nadir_ were statistically equivalent between the A‐STEP and CPET. These results confirm that the incremental A‐STEP is a maximal exercise test that can be used as a surrogate to CPET in a regular outpatient clinic room to measure exercise capacity facilitating modern infection control requirements. At the same clinic visit, exercise can be prescribed in real time for adults with CF across a range of age, height, body mass, lung function, and exercise fitness using the exercise data recorded. This information can be communicated to the relevant members of the CF team for treatment planning.

Oxygen uptake at the anaerobic threshold (VO_2@AT_) occurred significantly earlier during CPET compared to the A‐STEP, most likely attributable to the differences in exercise kinetics between the two modalities. Early reliance on anaerobic glycolysis to meet energy demands during loaded cycling would result in an increase in ventilatory drive, demonstrated by the significantly higher VE_Max_ for CPET versus the A‐STEP. In a recent study comparing CPET results from treadmill and cycle ergometer in triathletes, it was found that treadmill ergometry activates more muscle groups than cycle ergometry, and the VO_2AT_ and HR_AT_ also corroborated this being significantly higher in the treadmill versus cycle ergometry tests. The VO_2@AT_ is believed to be important as it corresponds to the threshold between moderate and high‐intensity exercise. At this point, the lack of sufficient supply to the exercising muscles necessitates glycolytic ATP production, and the accumulation of lactic acid occurs. Thus, in exercise at an intensity below the anaerobic threshold (AT), lactate remains at resting levels, while in high‐intensity exercise above AT, lactate rises until an elevated steady state is attained [20]. Treadmill and cycle ergometry are the two most commonly used CPET modalities in triathletes. Both have unique attributes including strengths and weaknesses. The cycle ergometer allows better ECG analysis due to fewer artifacts from upper body motion. HR_max_ is reported as similar or slightly higher in treadmill testing compared with cycle ergometry. HR_AT_ is often used to prescribe submaximal training loads, whereby training at the AT intensity improves the peak oxygen uptake. It has recently been demonstrated that a large volume of low‐intensity training (i.e., below the AT) is important for endurance athletes [21, 22].

The step height used in the 3MST and IST in the pediatric population was the same at 15 cm [3, 8]. In the A‐STEP, a step height of 20 cm was selected and standardized for all tests in order to limit variables. Altering step height depending on individual height and leg lengths was considered, but after trial of a 20 cm step with a number of pwCF across varying ages, lung function, and height during the development stage, a decision was made together with the pwCF that a 20‐cm step height was most suitable [9]. Further, this method mirrored the CPET protocol. There, cadence remained constant but intensity increased, while in the A‐STEP, the step height remained constant and the intensity increased through an incremental pace.

In CF care, the practice of exercise prescription following formal exercise testing was uncommon [6]. In confirming that the MST‐25 produced a similar exercise response to cycle ergometry, this shuttle test could be supported as a routine test for children in a center with space for a 10‐m track for walking/running. However, not all centers had a suitable space [4, 6]. In a center with regular‐sized outpatient clinic rooms, the A‐STEP would provide maximal exercise testing as a surrogate to testing with cycle ergometry in the laboratory. This would allow clinicians to routinely assess and monitor exercise capacity and accurately prescribe exercise training in real time in the clinical setting, while the frequently used MST‐15, 6MWT, and 3MST were all found to have ceiling effects with the 3MST also having a floor effect [3, 6]. The A‐STEP in this pilot study was found to be maximal with all subjects achieving at least one of the criteria denoting a maximal test without floor or ceiling effects.

The strength of our study is that it was successfully carried out in a small population of adults with CF with a wide range of age, height, weight, and lung function with highly comparable results. The incremental maximal A‐STEP test was externally paced with a metronome freely available online. The nonslip step was relatively inexpensive and commercially available. The test commenced at a slow cadence and increased incrementally to a faster pace with a speed equivalent to the 3MST at level 7 so that subjects had time to familiarize themselves and build a rhythm. At the higher levels (level 10), jogging was required to maintain the pace, while any remaining participants by level 16 had to run up and down the step in order to keep up with the cadence of the metronome at 48 beats per minute. At this pace, a certain level of coordination is required but pwCF usually achieves this level in the test. All the participants reached a VO_2MAX_ based on HR_MAX,_ mBorg, and RER in both the A‐STEP and CPET studies suggesting the methodology of the study was not a limiting factor.

The limitations of this study include that it was a single‐center study with a limited adult sample size. However, given the difficulties in recruiting for exercise studies, in a cohort of patients with an already high clinical burden, the close correlation of the data is reassuring, showing that the A‐STEP is a valid proxy of exercise capacity in pwCF. In addition, our previously published feasibility study recommended a future study comparing the A‐STEP with CPET in adults not on CFTR modulators; however, given the survival advantage seen with these agents, it would be unethical to withhold treatment. Nevertheless, it was possible to achieve a maximal test in all subjects, despite the progress in pharmaceutical treatments, maintaining the advantages of the A‐STEP in the new era of highly effective modulator therapy. We did not measure the effects of different step heights in shorter versus taller pwCF. The fitter pwCF thought the commencement pace was slow and thus took longer to complete the test as they needed to complete more levels to reach a VO_2peak_, although this was not statistically significant. A study in a larger population to verify these results is recommended, such as a study to determine the effects of different step heights based on height or ppFEV1 would be of benefit, while very fit pwCF and elite athletes starting at level 4 (24 beats per minute) would result in them potentially completing the test in the optimal time of 8–12 min. Studies investigating these different variables would add further nuance to the performance of the A‐STEP at the extremes of the normal demographic.

## 5. Conclusions

This pilot study may validate the A‐STEP as a portable and accessible surrogate to traditional maximal CPET using cycle ergometry, for exercise assessment in a broad range of adults with CF. A further study including very fit adults and children is warranted.

## Funding

The study was funded by the Alfred Foundation Small Projects Grant for funding for K5 silicon masks and consumables.

Open access publishing facilitated by Monash University, as part of the Wiley ‐ Monash University agreement via the Council of Australasian University Librarians.

## Disclosure

A preprint has previously been published [23].

## Conflicts of Interest

The authors declare no conflicts of interest.

## Data Availability

The data that support the findings of this study are available from the corresponding author upon reasonable request.
